# Physical Activity, Physical Fitness and the Sense of Coherence—Their Role in Body Acceptance among Polish Adolescents

**DOI:** 10.3390/ijerph17165791

**Published:** 2020-08-10

**Authors:** Ida Laudańska-Krzemińska, Jana Krzysztoszek, Mariusz Naczk, Ewa Gajewska

**Affiliations:** 1 Department of Physical Activity and Health Promotion Science, Poznan University of Physical Education, 61-871 Poznan, Poland; 2 Department of Didactics of Physical Activity, Poznan University of Physical Education, 61-871 Poznan, Poland; krzysztoszek@awf.poznan.pl; 3 Institute of Health Sciences, Collegium Medicum, University of Zielona Gora, 65-417 Zielona Gora, Poland; m.naczk@cm.uz.zgora.pl; 4 Department of Developmental Neurology, Poznan University of Medical Sciences, 60-355 Poznan, Poland; egajewska@ump.edu.pl

**Keywords:** body image, sense of coherence, physical activity, cardiorespiratory fitness, adolescents, body perception, body satisfaction

## Abstract

The aim of the study was to investigate the protective role of physical activity (PA) and other health-related bio-psycho components (physical fitness, body composition, body perception and the sense of coherence (SOC)) in body acceptance. We searched for gender differences in those relationships. We investigated 231 adolescents aged 13–16 years from an urban area in Poland. We conducted objective measurements of height, weight, fat% and relative value of minute oxygen consumption. Questionnaires for PA, SOC Body Figure Perception and body acceptance were applied. Linear regression was used for analyzing determinants of body acceptance. We found that more physically active girls reported a less slim ideal vision of their figure (*p* < 0.05). Physical fitness is a better predictor of body acceptance than physical activity. In the final model, the sense of coherence, body mass index (BMI), and gender (being a boy) were also predictors of body acceptance (F(6,92) = 13.084, *p* < 0.0001). Gender differences were discussed in the present study. Fitness enhancing physical activity should be recommended for adolescents to achieve the protective psychosocial effect especially among girls. Physical activity on a daily basis brings positive results in a more adequate and reasonable body assessment and it can play a protective role in terms of mental wellbeing. Body satisfaction varies between genders and it is a more sensitive issue among girls than boys during adolescence.

## 1. Introduction

It is recommended that young people should engage in moderate-to-vigorous intensity physical activity (MVPA) for an average of at least 60 min per day throughout the week. This can include all forms of activity such as physical education, active travel, after-school activities, play and sports [1]. It is estimated that 80% of teenagers worldwide are not sufficiently active [2]. A recent study in eight European countries showed that only 9% of girls and 26% of boys were physically active and performed ≥60 min/day of MVPA [3]. According to the Global Matrix 3.0 report [4], the overall rate of PA of children and adolescents is low in the vast majority of the 49 countries that were assessed. Mazur [5] states that only 17.2% of 11-to-15-year-olds in Poland meet the requirement of 1 h of MVPA per day. There has been a substantial decline in the cardiorespiratory fitness level among adolescents since 1981, which is suggestive of a meaningful decline in the health of the population [6].

Physical activity has been found to have a positive impact on both physical and psychological health [7,8]. Júdice et al. suggested that encouraging young people to engage in more moderate- and vigorous-intensity physical activity and reduce their sedentary time may have beneficial effects on their health-related fitness [9]. Also, physical fitness is an important predictor of health in youth [10,11]. The concept of the body image brings together the idea of the physical form with the more subjective perception of a person’s size. Body image can roughly be characterized as the conscious, predominantly visual, mental representation of one’s own body, which in turn provides a basis upon which perceptual, cognitive, and affective attitudes toward the body are assigned [12,13]. However, it is important to note that the current literature largely concurs on the use of the term of body image as a measure of body satisfaction—in that body image as an outcome measure is interpreted as the degree to which individuals are satisfied with various aspects of themselves that are influenced by the visual aspect of their bodies (e.g., appraisal of body shape information) [14]. Body satisfaction is usually measured in adolescents by asking them to rate their level of satisfaction with aspects of their body, such as height, weight, shape, waist, build, face, and specific body parts [15,16,17]. Body mass index (BMI) represents the physiological aspect of body changes during adolescence, while the body image perception represents the cognitive component, and body image satisfaction corresponds to the emotional component of the body image. Moreover, these domains are also related to behavioral components, dieting or exercising, that contribute to the control of weight and body image [18]. Body dissatisfaction is reported by up to one-third and every other adolescent boy and girl, respectively [19,20]. Duchesne et al. [21] noted that as many as 63.5% of adolescent girls report poor body image. One factor contributing to poor body image is that girls learn from their families, friends, and other sources, such as the media, that thinness is desirable [22]. What is of concern is that girls’ negative perceptions of their body weight, shape, or size place them at a high risk for low self-esteem, decreased self-worth, poor self-concept, and negative affect [23]. Several studies have suggested that increased physical activity participation is associated with a more positive body image among adolescent girls [24,25,26].

Altıntaş et al. [27] found that self-reported PA was not correlated with body image satisfaction among adolescent girls. In randomized controlled trials with overweight and obese adolescents, body image improved following participation in the PA intervention offered in each study [28,29]. Physical activity intervention can be cautiously regarded as a potentially effective option regarding body image-related outcomes [14]. Body image, rather than BMI, is important in undertaking physical activity in adolescents and should be considered when preparing programs aimed at improving physical activity [25].

Assisting girls to attain and sustain healthy weight remains critical for helping them to achieve and maintain a positive body image. The high negative correlation between cardiovascular fitness and the percent of body fat indicates that interventions to promote a positive body image among girls may need to include strategies to increase PA as a means to improve their cardiovascular (CV) fitness, thereby reducing their percent of body fat. Evidence has shown that the increased muscle tone, strength, physical competence or fitness, and reduced body size, resulting from increased PA can help to improve body image perceptions [30]. However, studies show that a significant number of adolescents attempting to lose weight also use physical activity or exercising strategies as well [31]. The body fat percentage is directly related to the body image discrepancy, while PA and cardiovascular fitness have an indirect effect on the body image discrepancy via the body fat percentage [32].

According to Antonovsky [33] the concept of the health continuum, the sense of coherence (SOC), is a good predictor of mental health and psycho-social wellbeing also during adolescence. The sense of coherence is considered to be a key variable in the study of individual differences on health and coping with issues. Studies confirm that the SOC can be considered as a factor strengthening resilience and developing a positive subjective state of health. A strong SOC predicts reduced stress and decreased internalizing or/and externalizing problems. A systematic review showed that adolescents’ sense of coherence was related to health in terms of the quality of life, health behavior, mental health and family relationships [34]. Accordingly, results of various studies showed that adolescents with higher SOC reported a healthier lifestyle, a better quality of life, and well-being [35] or less body dissatisfaction [36,37] and higher physical activity [38]. SOC is also strongly and positively related to self-esteem among adolescents and it is higher among boys than girls [39]. It was also shown that SOC was a good detector for anxiety and depression among adolescent girls [40].

Children with higher BMI may feel discriminated against due to their image, which could lead them to presenting long-term internalizing symptoms. This is related to an increased awareness and internalization of social attitudes around weight throughout childhood, which is manifested by greater psychological problems in adolescents with a worse body image [41]. Due to the fact that girls have more body image concerns than boys [17], understanding factors uniquely related to the girls’ body image discrepancy may be important for developing targeted interventions to address body-related issues and prevent adverse health-related sequelae in this population [22].

The aim of the study was to investigate the protective role of physical activity and other health-related bio-psycho components (physical fitness, body composition, body perception and the sense of coherence) for body acceptance, as well as the relationships between them in relation to body acceptance. We examined for gender differences in those relationships.

## 2. Materials and Methods

### 2.1. Participants and Measurements

The study involved 231 adolescents (123 girls and 108 boys) aged 13 to 16 (M = 14.8; SD = 1.1). The respondents came from an urban area in Poland. Adolescents were recruited from schools in cities (Gorzów Wielkopolski and Poznań) with a population of over 100,000 by their teachers. The research was carried out in the laboratory of the University of Physical Education by PhD students and laboratory staff, and the questionnaire was completed in the classroom on paper between 2018 and 2019. Body mass and height data were collected with the use of anthropological instruments in the laboratory. For each participant, BMI was calculated according to the formula: weight/height^2^ (kg/m^2^). World Health Organization (WHO) standards were used to define adolescents underweight and overweight [42]. Children aged 6–19 years are overweight with excess BMI over + 1 SD and underweight under -2 SD. Detailed data are presented in Table 1.

In all subjects, the body fat was assessed by means of bioimpedance using the Tanita MC-980MA multi-frequency analyzer (Tanita Corporation, Tokyo, Japan). The analysis of body composition using bioimpedance is precise enough to determine body composition and it is recommended for epidemiological purposes [43]. The participants were asked to maintain a normal state of hydration and they were not allowed to exercise for 12 h preceding the measurements. Measurements were conducted in the morning according to the manufacturer’s guidelines. The results were given as a percentage of total body weight (%).

The maximal oxygen uptake was determined during a running test on the HP Cosmos treadmill (h/p/cosmos, Nussdorf—Traunstein, Germany). Breath-by-breath oxygen uptake was continuously recorded using the START 2000 ergospirometer (MES, Skawina, Poland). The test was incremental; all participants commenced the test at the running speed of 7 km/h, which was increased by 1 km/h every 2 min until maximum individual loads were obtained or until the subject refused to continue the activity. The three parameters for task termination were as follows: the respiratory exchange ratio > 1.1, occurrence of the oxygen consumption plateau despite increases in the running speed and the achievement of the maximum heart rate in relation to the age of the participant. When two of the three criteria mentioned above were met and the participant was not able to run because of fatigue, the test was completed. The relative value of one-minute maximal oxygen uptake (VO_2_max (mL/kg/min)) was used as a parameter to characterize the respiratory system and the circulatory system functions.

The level of moderate-to-vigorous physical activity (MVPA) was determined with the Physical Activity Screening Measure [44]. It corresponds to the average number of days per week with at least 60 min spent undertaking various forms of PA during which, in the participants’ subjective opinion, their heart rates increased, and they experienced a feeling of shortness of breath (higher breathing frequency). The scores ranged from 0 to 7 (days/week). We divided participants on two groups: active (at least 6 days/week completing the recommendation) and non-active (see Table 1).

The sense of coherence was measured using the SOC-13 questionnaire [45,46]. The questionnaire contains 13 statements from the adult version (SOC-29), which are rated on a five-point Likert scale. The SOC-13 score ranged from 13 to 65. The SOC-13 scale has three components: Meaningfulness (4 items), Comprehensibility (5 items), and Manageability (4 items). In the present study Cronbach’s α for general scale was 0.81 and for each dimension as follows: Meaningfulness α = 0.70, Comprehensibility α = 0.71 and Manageability α = 0.89.

Body acceptance was measured using a single-item questionnaire: “*To what extent do you accept your body?*”, which is rated in the seven-point Likert scale, from “*not at all accepted*” to “*fully accepted*”. Body perception was measured using the Body Figure Perception (BFP) scale modified by Collins [47], which is a visible measure of how an individual perceives his or her own physical appearance. Each figure presents schematic silhouettes of seven girls and seven boys, ranging from extreme thinness to extreme obesity. Participants were asked to self-select the silhouette that best indicates his or her current body size and the silhouette that reflects his or her ideal body size.

### 2.2. Ethics Approval and Consent to Participate

The study protocol was approved by the Local Bioethics Committee of the Karol Marcinkowski University of Medical Sciences in Poznan (decision number 710/17). Moreover, written consent for all activities included in the experiment was obtained from the adolescents’ parents.

### 2.3. Statistics

To evaluate the differences between the two compared groups (boys and girls, non-active and active respondence) the Mann–Whitney U-test was used. Linear regression was used for analyzing determinants of body acceptance. Furthermore, the ŋ^2^ effect size coefficient was calculated. The effect size coefficient was interpreted as follows: small effect size ŋ^2^ = 0.01–0.059, medium effect size ŋ^2^ = 0.06–0.139, large effect size ŋ^2^ ≥ 0.14. A significance level of *p* < 0.05 was assumed for all tests [48]. The calculations were made using the Statistica 13.3. software (StatSoft, Krakow, Poland).

## 3. Results

The results show that girls have lower body acceptance (large effect size) and they indicate a thinner body as an ideal figure more frequently than boys (medium effect size). Then, boys manifest lower body fat percentages and higher physical fitness (both large effect size). In Table 2 we present gender differences among the analyzed parameters: body acceptance, assessing body figure at present and the ideal one, sense of coherence and their dimensions (Meaningfulness, Comprehensibility, Manageability), BMI, body fat percentages, MVPA and the relative value of minute oxygen consumption.

In the next step we analyzed differences between surveyed adolescents who are active or non-active (Table 3). Physically active girls and boys had a higher sense of coherence (general and in Comprehensibility and Manageability dimensions). They also had higher maximum oxygen uptake and MVPA. The obtained effect sizes are small, except MVPA (large effect) which was the basis for the division into active/non-active groups.

The differences in body acceptance, current body figure and the ideal one analyzed among the surveyed girls showed that non-active girls pointed to a slimmer ideal vision of their figure than those active ones (*p* < 0.05, ŋ^2^ = 0.032—small effect size) and assessed their body as a slimmer (*p* < 0.05, ŋ^2^ = 0.034—small effect size). The analysis showed that active girls had lower fat percentage than non-active ones (*p* < 0.05, ŋ^2^ = 0.111—medium effect size). We did not find such differences among boys.

The linear regression analysis performed for body acceptance as a dependent variable indicates that physical, physiological and psychological variables have a significant effect on body acceptance (*p* < 0.001) and explain 42% of variance in Model 1 (Table 4). The results show that physical fitness is a better predictor of body acceptance than MVPA. In Model 1, the sense of coherence, BMI and gender are also significant predictors of body acceptance (F(6,92) = 13.084, *p* < 0.0001). Average body acceptance increases due to higher sense of coherence, lower BMI, higher physical fitness and the fact of being a boy. When we added two more factors which describe the current perception of the body and the ideal pattern, it turned out that apart from gender and sense of coherence, self-esteem of the body corresponds to the body acceptance. Model 2 explains 45% of the variance (F(8,90) = 11.177, *p* < 0.0001). Average body increases due to higher sense of coherence, lower perception of current body size and the fact of being a boy.

## 4. Discussion

It is widely acknowledged that the health benefits of participation in PA are not limited to physical health but also incorporate mental components [49,50]. In our study, we found that more active youth showed a higher level of SOC—in general and in comprehensibility and manageability dimensions. It is also indirectly confirmed by the study of Dyremyhr et al. [51], that sport participation is correlated with wellbeing and by the study of Guddal et al. [52], that higher levels of PA are favorably associated with psychical health, especially with self-esteem and life satisfaction throughout adolescence, as well as with reduced likelihood of psychological distress in senior high school students. Kołoło et al. [53] proved the existence of a curvilinear relationship between adolescent physical activity levels and self-esteem in terms of appearance and body image. Another study shows that physical exercise was most strongly associated with the perception of health, even when several social and psychological risk factors were included in the analysis. SOC and variables of social support were also of importance [54].

It is also worthwhile to mention that the mechanism and determinants of body image among adolescent boys and girls differ in some aspects [55,56,57,58]. Boys tend to be more dissatisfied when their BMI is below or above average; girls tend to be dissatisfied when their BMI is average or above average [57,58]. Most of the studies report that ideal body image for boys relate to muscular fitness while for girls it means to be thinner and slimmer [58]. This is in line with our results.

The systematic review of the psychological and social benefits of participation in sport for children and adolescents shows higher self-esteem, better social skills, higher confidence and higher competence amongst sport participants than non-sport participants (many studies did not distinguish between sport and other categories of PA) [59]. The study of Lawler et al. [60] who examined differences in sport participation has shown that adolescent girls who participate in organized team or individual sport demonstrate higher levels of intrinsic motivation, competence, relatedness, autonomy and autonomy support relative to those less active ones in organized PA or non-organized PA and non-participants.

Another factor which could support adolescent’s sense of coherence and which is also related to PA is social support. Korcz et al. [61] reported that boys who participate in sport often got feedback from their friends that they were doing well at PA and they had significantly more friends due to participating in PA or playing sport games. Guddal et al. [52] emphasize the importance of social and PA with peers, which may be particularly beneficial for older adolescents, helping them to distract from depressive thoughts and to reduce the sense of isolation. In addition, our research showed that girls aged 14-16 have significantly lower self-esteem than boys, they consider themselves as more obese, although the BMI level in both sexes was basically the same. Furthermore, clear sex differences were found with regards to problems related to body image in a study by Ramos et al. [41]. The results showed that girls, more often than boys, see themselves as fat, feel dissatisfied with their body image, and got on a diet to lose weight/volume. Similarly, Kołoło et al. [53] note that insufficient physical activity was also found among those adolescents with a negative body image or a negative perception of their body weight. We suppose that one of the factors explaining the obtained result may be lower sports activity of girls compared to boys, which is a fairly common phenomenon especially at puberty and adolescents [53,62]. Furthermore, physical and sport activity is one of the significant predictors of higher self-esteem [53]. A study by Hansen et al. [63] study showed that young people involved in sports have higher rates of self-knowledge and emotional regulation. This could explain the difference in self-esteem of young people. Moreover, Guddal et al. [52] found that participation in sports was significantly associated with reduced odds of low self-esteem for girls, both in junior high school and senior high school.

We found that the ideal version of a body for the examined inactive girls is slimmer than for active ones. Similarly, Dyremyhr et al. [51] stated that girls who did exercise irregularly tried to lose weight more than those exercising regularly. At the same time, a lot of adolescent girls are involved in PA because of the motivation to improve their body shape [64]. Studies also show that the vast majority of girls participate in PA irregularly, and some of them set up unrealistic goals to achieve an ideal body shape while their body weight is absolutely normal. Therefore, it might be one of the reasons to abandon exercise during adolescence, as they experience frustration related to their body image [60]. This can indirectly be proven by low body acceptance and low self-esteem related to the body image of inactive girls during adolescence. In our study we also confirmed, that the vision of the ideal figure for non-active adolescent girls is slimmer than the recommended BMI values.

Homan and Tylka [65] see two mechanisms of this phenomenon. They showed that regular exercise is strongly and directly related to higher satisfaction with what the body can do physically. Secondly, it is plausible that girls who exercise primarily for weight loss or to change the shape of their bodies interpret their experience differently from those who are primarily motivated by other reasons. The present study supports this idea. Inactive girls undertaking irregular physical activity are likely to focus on factors such as the number of calories burned, immediate reduction in body weight, or visible changes in appearance. Concern with these issues may overshadow positive emotions such as satisfaction that can arise from completing a physical challenge or appreciating the body’s capabilities. Low or no effectiveness resulting from irregular activity and lack of perception of pleasure in doing PA has a demotivating effect for its continuation. In turn, active girls, who exercise for reasons unrelated to appearance (which presumably would include health or enjoyment, better mood) may be more likely to attend to the sensations that typically accompany exercise. They are more satisfied with their bodies, have a higher self-esteem probably due to the fact that they notice and appreciate what their bodies can do. This may help them to accept their bodies despite cultural messages about the desirability of thinness.

In addition, our study shows that inactive girls not only wanted to be slimmer, but rated themselves as slimmer than active girls, despite the fact that the objective indicator (fat%) was higher for them that for active girls. This may be another demotivating factor for physical activity. As stated by Kołoło et al. [53], the main motivating factor for girls to participate in physical activity appears to come from dissatisfaction with their appearance. If inactive girls rate the active ones as less attractive, i.e., less lean than themselves, and this indicator is extremely important for them, it demotivates them to undertake physical activity and motivates them to look for another way to achieve the goal, striving for a slimmer body shape (e.g., introducing nutritional changes), which active girls do not care so much about.

Another study [37] shows that physical fitness training improves not only the body image but also the sense of coherence of young women. In our study, we found that SOC has the most protective role in accepting the body, and we confirmed the importance of undertaking sports activities. This is especially important for girls during this developmental period. This is a period of a significant decrease in the girls’ physical activity, and the protective role is significantly played by physical activity improving physical fitness parameters (e.g., VO_2_max). Therefore, health promotion initiatives for adolescents should promote intense physical and sports activities and should put an emphasis on building self-esteem and body satisfaction, which is also noticed by other authors [65]. It could also be worth using a salutogenic approach in Physical Education, which connects mental and physical recourses during classes [66].

Team sports also seem to be a good solution. Guddal et al. [52] proves that team sports give the greatest psychological benefits for girls at puberty. A greater feeling of belonging and affiliation arising from high quality relationships with teammates may also account for higher levels of intrinsic motivation among youth represented by profiles of team sport participation relative to their individual sport, and non-participating counterparts [60].

Limitations and strength of the study. The adolescents tested in this study are from an urban area of Poland, therefore they do not represent all adolescents from Poland. Moreover, the sample of 231 adolescents is acceptable but still small, which is a limitation of the study. The influence of many factors on the body acceptance were tested; we tested both influence of physiological factors and indicators included in the questionnaires. This provided a comprehensive way to assess the impact of various factors on the body acceptance.

## 5. Conclusions

Fitness enhancing physical activity should be recommended for adolescents to get the protective psychosocial effect especially among girls. Physical activity on a daily basis brings results in a more adequate and reasonable body assessment and it can play a protective role in terms of mental wellbeing. Body satisfaction varies depending on gender and it is a more sensitive issue during adolescence among girls than boys. We observed differences between active and inactive girls in terms of body satisfaction, where PA plays a protective role for emotional wellbeing, and not only allows to meet the recommended MVPA level in terms of health. The salutogenic approach for the promotion of physical activity and implementation of physical education may be an effective basis for the prevention of mental health problems. Taking into account the results of this research, it is advisable to ensure high quality preventive health care for schoolchildren by public health care institutions. Health-oriented behaviors should be promoted, in particular individual responsibility for one’s own health should be encouraged, and greater insight should be given before taking the decision to exempt young people from compulsory physical education classes, which are sometimes the only form of physical activity. It is also extremely important to involve the doctor’s authority in the implementation of health education for students and their parents, making them aware of the beneficial consequences of physical activity not only in terms of physical health, but also in the context of the mental condition of young people and maintaining psychosocial benefits.

## Figures and Tables

**Table 1:** Characteristics of the surveyed adolescents.

Characteristics	N	Age (years)	BMI (kg/m^2^)	BMI Categories			Non-Active	Active
		Mean (SD)	Mean (SD)	Underweight n (%)	Normal n (%)	Overweight n (%)	N (%)	N (%)
Girls	123	14.7 (1.1)	20.7 (2.7)	3 (2.5)	95 (77.2)	25 (20.3)	79 (64.2)	44 (35.8)
Boys	108	14.9 (1.1)	21.2 (3.6)	4 (3.7)	72 (66.7)	32 (29.6)	73 (67.5)	35 (32.5)
All	231	14.8 (1.1)	20.9 (3.2)	7 (3.0)	167 (72.3)	57 (24.7)	152 (65.8)	79 (34.2)

Note: N, number; SD, standard deviation.

**Table 2:** Gender differences in the analyzed characteristics.

Characteristics	All (N = 231)		Girls (N = 123)		Boys (N = 108)		*p*-Value	ŋ^2^
	Mdn	M (SD)	Mdn	M (SD)	Mdn	M (SD)		
Body acceptance (pts.)	5.0	4.8 (1.5)	5.0	4.3 (1.5)	5.0	5.4 (1.2)	**<0.001**	0.141
BFP current (pts.)	4.0	3.8 (1.0)	4.0	3.9 (1.0)	3.5	3.8 (1.0)	0.446	0.002
BFP ideal (pts.)	3.5	3.4 (0.7)	3.0	3.2 (0.7)	3.8	3.6 (0.6)	**<0.001**	0.075
SOC-13 (pts.)	42.5	40.7 (7.2)	41.0	39.6 (8.6)	43.0	42.0 (4.8)	0.274	0.011
Comp (pts.)	16.0	15.7 (3.3)	15.0	15.0 (3.7)	17.0	16.4 (2.4)	0.067	0.031
Mana (pts.)	13.0	12.7 (2.7)	13.0	12.5 (3.0)	13.0	12.8 (2.3)	0.858	0.000
Mean (pts.)	13.0	12.4 (2.3)	12.0	12.1 (2.6)	13.0	12.8 (2.0)	0.364	0.007
BMI (m/kg^2^)	21.0	20.9 (3.2)	20.4	20.7 (2.7)	20.7	21.2 (3.6)	0.460	0.002
Fat (%)	22.8	22.1 (6.0)	25.2	26.0 (3.5)	17.0	17.5 (5.0)	**<0.001**	0.512
VO_2_max (mL/min/kg)	46.7	46.8 (10.2)	43.4	42.0 (8.3)	51.5	51.8 (9.7)	**<0.001**	0.213
MVPA (days/week)	5.0	4.7 (1.8)	5.0	4.7 (1.8)	5.00	4.6 (1.8)	0.986	0.000

Note: bold, *p* < 0.05; Mdn, median; pts., points; M, mean; SD, standard deviation; BFP, Body Figure Perception; Comp, Comprehensibility; Mana, Manageability; Mean, Meaningfulness.

**Table 3:** Differences between active and non-active girls and boys (N = 231).

Variables	Non-Active (N = 152)			Active (N = 79)			All		Girls		Boys	
	All	Girls	Boys	All	Girls	Boys						
	M (SD	M (SD	M (SD)	M (SD	M (SD	M (SD)	*p*-Value	ŋ^2^	*p*-Value	ŋ^2^	*p*-Value	ŋ^2^
Body acceptance (pts.)	4.7 (1.5)	4.2 (1.5)	5.3 (1.3)	4.9 (1.5)	4.3 (1.4)	5.6 (1.1)	0.446	0.002	0.799	0.001	0.275	0.01
BFP current (pts.)	3.8 (1.1)	3.8 (1.1)	3.8 (1.0)	3.9 (1.0)	4.1 (1.0)	3.7 (0.9)	0.362	0.004	**0.044**	0.032	0.346	0.008
BFP ideal (pts.)	3.4 (0.8)	3.1 (0.7)	3.6 (0.7)	3.5 (0.6)	3.4 (0.7)	3.6 (0.5)	0.391	0.003	**0.038**	0.034	0.146	0.018
SOC-13 (pts.)	39.2 (7.9)	37.3 (9.4)	41.4 (5.1)	42.3 (6.0)	42.0 (7.1)	42.6 (4.5)	**0.049**	0.037	0.086	0.052	0.320	0.02
Comp (pts.)	14.8 (3.6)	13.7 (3.9)	16.1 (2.8)	16.6 (2.5)	16.4 (3.0)	16.8 (1.9)	**0.016**	0.054	**0.012**	0.109	0.328	0.02
Mana (pts.)	12.1 (2.9)	12.0 (3.1)	12.2 (2.6)	13.2 (2.4)	13.1 (2.9)	13.4 (1.9)	**0.042**	0.038	0.201	0.029	0.111	0.052
Mean (pts.)	12.3 (2.5)	11.6 (2.8)	13.1 (2.0)	12.5 (2.1)	12.5 (2.3)	12.4 (1.9)	0.555	0.003	0.168	0.032	0.413	0.013
BMI (m/kg^2^)	21.0 (3.4)	20.6 (2.8)	21.3 (3.9)	20.9 (2.7)	20.9 (2.5)	21.0 (3.0)	0.742	0.000	0.611	0.002	0.969	0.000
Fat (%)	22.9 (6.6)	27.4 (3.8)	17.9 (5.4)	21.3 (5.1)	24.6 (2.5)	17.1 (4.5)	0.233	0.014	**0.012**	0.111	0.583	0.007
VO_2_max (mL/min/kg)	44.9 (12.2)	38.1 (8.1)	51.7 (11.9)	48.8 (7.2)	46.0 (6.6)	51.9 (6.7)	**0.021**	0.053	**0.001**	0.22	0.946	0.000
MVPA (days/week)	3.8 (1.5)	3.7 (1.5)	3.8 (1.6)	6.4 (0.4	6.3 (0.4)	6.4 (0.4)	**<0.001**	0.741	**<0.001**	0.684	**<0.001**	0.651

Note: bold, *p* < 0.05; M, mean; SD, standard deviation; pts., points; BFP, Body Figure Perception; Comp, Comprehensibility; Mana, Manageability; Mean, Meaningfulness; MVPA, moderate-to-vigorous physical activity.

**Table 4:** Results of linear regression for body acceptance.

Predictors	Standardized *β* Beta	*R* ^2^	Change in *R*^2^	Change in *F*	*F*(df)
**Model 1**		0.425	0.425	1.109 ***	13.084 (6.92)
Gender (0-boys, 1-girls)	−0.426 *				
SOC-13	0.362 ***				
BMI	−0.297 *				
Fat %	0.193				
VO_2_max	0.228^m^				
MVPA	0.049				
**Model 2**		0.454	0.029	1.081***	11.177 (8.90)
Gender (0-boys, 1-girls)	−0.422 **				
SOC-13	0.347 ***				
BMI	−0.163				
Fat %	0.216				
VO_2_max	0.149				
MVPA	0.036				
BFP current	−0.273 *				
BFP ideal	0.112				

Note: m, marginal effect *p* value < 0.08; * *p* value < 0.05; ** *p* value < 0.01; *** *p* value < 0.001; BFP, Body Figure Perception; SOC-13, sense of coherence.
